# A Discourse on Self-Limited Diseases

**Published:** 1837-01

**Authors:** 


ART. III.—*A Discourse on Self-limited Diseases, delivered before the Massachusetts' Medical Society, at their Annual Meeting, May* 27, 1835. By JACOB BIGELOW, M.D., Professor of Materia Medica in Harvard University.—*Boston,* 1835. 8vo. pp. 48.

THERE is nothing which gives us more pleasure, in the course of our editorial duty, than to meet with such frequent instances as come before us of the liberal and philosophical spirit with which medical science is cultivated by the physicians and surgeons of the United States.

This discourse, in itself an illustration of this remark, begins by a brief and graceful allusion to the recent deaths of three valued members of the Society before which it was delivered. The author then proceeds to make some just and sensible observations on the imperfections of the healing art, some of which he attributes "to the apparent fact, that certain morbid processes in the human body have a definite and necessary career, from which they are not to be diverted by any known agents with which it is in our power to oppose them." It is to these that Dr. Bigelow applies the term *self-limited.* For examples he refers to vaccinia, varicella, and the salivation produced by mercury: of these he says he knows of no medical practice which can divert them from their course or hasten their termination. Dr. Bigelow also refers to hooping-cough, measles, scarlet fever, small-pox, erysipelas, and (with some exceptions) typhus fever; and he thinks it probable that other examples might be taken from other forms of fever and from the acute inflammations. Epilepsy and angina pectoris are added to the number, but gout is excepted. Other diseases which come on in paroxysms, as mania, asthma when dependent on pulmonary emphysema, gravel, and the symptoms of ascarides, are enumerated under the same head. Metastatic diseases furnish other instances; such as certain cutaneous affections which alternate with irritations of some internal organ; and, above all, acute rheumatism. Structural lesions and pestilential diseases are also alluded to.

It must be confessed that there is sufficient ground for reflections of this nature to cast a gloom over the mind of a medical practitioner: the heads here enumerated comprehend a vast proportion of human maladies; but, on the other hand, Dr. Bigelow very properly reminds us of the many instances, daily witnessed, of pains relieved, spasms controlled, inflammations checked, and diseased associations broken up. He speaks of the great achievements of limiting the ravages of variola and of syphilis; and, if he had thought it necessary, he might have dwelt with justifiable complacency on the extensive benefit derived from medicine in the slighter disturbances of the organs which are termed functional; those, for instance, of the stomach and bowels; as well as on the extensive power possessed of giving relief even in diseases the most incurable.

The proper use to be made of such considerations is, to regard them as inducements to a candid examination of the actual effect of remedial measures in every case; to a careful and zealous employment even of the means of palliation and relief; and to an anxious and industrious search after the means of increasing the powers of medicine. On all these points Dr. Bigelow's discourse contains excellent observations, which bear the impression of much experience, mature reflection, and an enlightened understanding.

